# P-208. The Quiet Danger of Dengue in the Elderly: Three Years of Experience in a Tertiary Teaching Hospital

**DOI:** 10.1093/ofid/ofaf695.430

**Published:** 2026-01-11

**Authors:** Ann S Sánchez-Marmolejos, Rita A Rojas-Fermín, Karla Marie Disla-Pineda, Lia Chaddy-Baéz, Jharasy Jiménez, Anel E Guzmán-Marte, Yeison Reyes-Burgos

**Affiliations:** Hospital General de la Plaza de la Salud, Distrito Nacional, Distrito Nacional, Dominican Republic; Hospital General De La Plaza De La Salud, Distrito Nacional, Distrito Nacional, Dominican Republic; Hospital General de la Plaza de la Salud, Distrito Nacional, Distrito Nacional, Dominican Republic; Hospital General De La Plaza De La Salud, Distrito Nacional, Distrito Nacional, Dominican Republic; Hospital General De La Plaza De La Salud, Distrito Nacional, Distrito Nacional, Dominican Republic; Hospital General De La Plaza De La Salud, Distrito Nacional, Distrito Nacional, Dominican Republic; Hospital General Plaza de la Salud, santo domingo, Distrito Nacional, Dominican Republic

## Abstract

**Background:**

Dengue is a dynamic disease and recurrent public health challenge in the Dominican Republic, with DENV-1, DENV-2, and DENV-3 circulating between 2022 and 2024. The re-emergence of DENV-3 after years of absence increased population vulnerability. Older adults face higher risk due to immunosenescence, more comorbidities, and atypical symptoms. This study aimed to characterize the clinical, epidemiological, and laboratory profile of elderly patients hospitalized with dengue and compare them with younger adults to identify differences relevant to clinical care.

**Figure:**
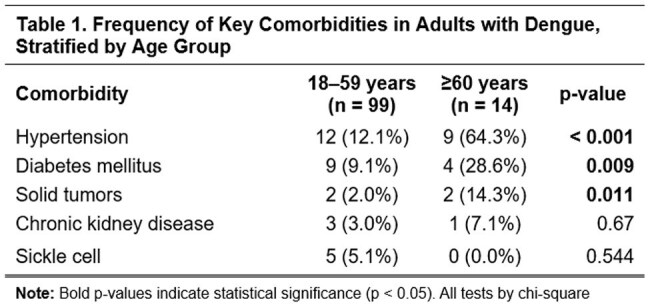

**Figure:**
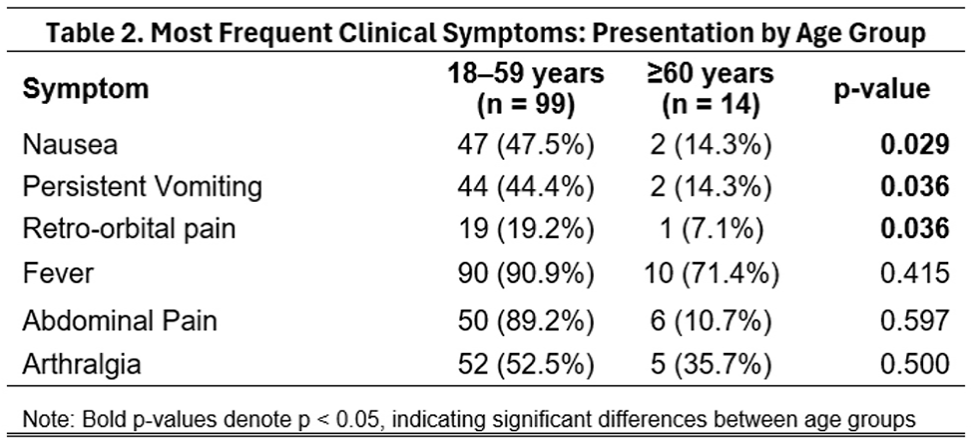

**Methods:**

We conducted a retrospective analysis of 113 adults with laboratory-confirmed dengue hospitalized at Hospital General de la Plaza de la Salud from January 2022 to March 2024. Patients were stratified into two groups: Elderly (≥60 years, n=14) and younger adults (18–59 years, n=99). We compared comorbidities, symptoms, laboratory values at admission, WHO 2009 severity classification, and clinical outcomes. Associations were evaluated using chi-square tests (p< 0.05).

**Figure:**
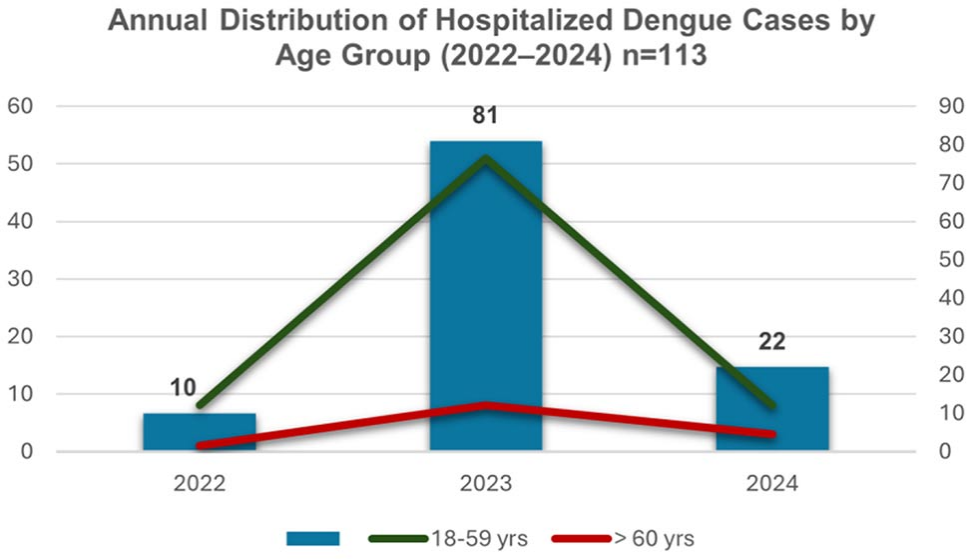

**Figure:**
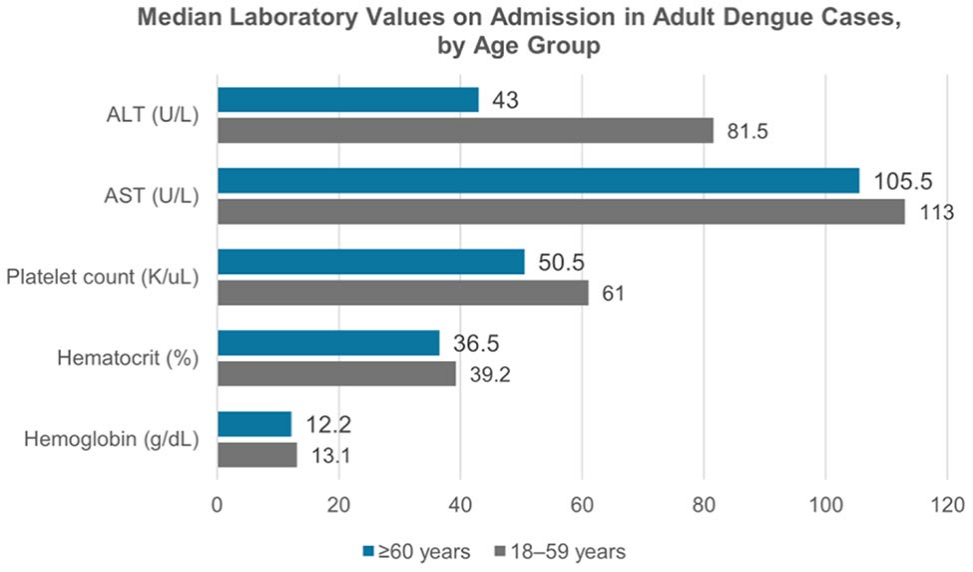

**Results:**

Of the total cohort, 12.4% were ≥60 years (median age: 64.5; range: 60–86). At admission, 85.7% were classified as dengue with warning signs and 14.3% as severe dengue. The most common warning signs were abdominal pain (10.7%) and persistent vomiting (14.3%). Elderly had more comorbidities, especially hypertension (64.3%, p< 0.001), diabetes (28.6%, p=0.009), and solid tumors (14.3%, p=0.011). They showed fewer typical symptoms like nausea (p=0.029), vomiting (p=0.036), and retro-ocular pain (p=0.036). Lab findings included lower leukocyte counts, hemoglobin, hematocrit, and platelets (median: 50.5 K/uL; range: 15-109), with higher AST levels (median: 105.5 U/L; range: 19–1191). Three required ICU care; one died.

**Conclusion:**

In elderly patients, dengue may present atypical manifestations. They may not have classic symptoms, but the disease can still become serious. Because older adults often have more comorbidities, new complications can go unnoticed until they become severe. As the population ages, healthcare workers must stay alert and recognize that dengue in elderly patients may appear in a quiet way but still carry high risks. Early attention can help prevent complications and save lives in this vulnerable group.

**Disclosures:**

Rita A. Rojas-Fermín, MD, GSK: Honoraria

